# The Effects of 12 Weeks Colostrum Milk Supplementation on the Expression Levels of Pro-Inflammatory Mediators and Metabolic Changes among Older Adults: Findings from the Biomarkers and Untargeted Metabolomic Analysis

**DOI:** 10.3390/nu15143184

**Published:** 2023-07-18

**Authors:** Theng Choon Ooi, Azizan Ahmad, Nor Fadilah Rajab, Razinah Sharif

**Affiliations:** 1 Centre for Healthy Ageing and Wellness, Faculty of Health Science, Universiti Kebangsaan Malaysia, Kuala Lumpur 50300, Malaysia; ooithengchoon@ukm.edu.my (T.C.O.); nfadilah@ukm.edu.my (N.F.R.); 2 School of Chemical Science and Food Technology, Universiti Kebangsaan Malaysia, Bangi 43600, Malaysia; azizan@ukm.edu.my

**Keywords:** biomarkers, colostrum, inflammation, metabolomics, older adults

## Abstract

Senescence is a normal biological process that is accompanied with a series of deteriorations in physiological function. This study aimed to investigate the effects of bovine colostrum milk supplementation on metabolic changes and the expression of various biomarkers on inflammation, antioxidant and oxidative damage, nutrient metabolism, and genomic stability among older adults. Older adults (50–69 years old) who participated in the 12-week randomized, double-blinded, placebo-controlled trial were instructed to consume the IgCo bovine colostrum-enriched skim milk or regular skim milk (placebo) twice daily. Following 12 weeks of intervention, participants in the intervention group had lower expression levels in pro-inflammatory mediators (CRP, IL-6, and TNF-α), with significant (*p* < 0.05) interaction effects of the group and time observed. However, no significant interaction effect was observed in the vitamin D, telomerase, 8-OHdG, MDA, and SOD activities. UPLC-MS-based untargeted metabolomics analysis revealed that 22 metabolites were upregulated and 11 were downregulated in the intervention group compared to the placebo group. Glycerophospholipid metabolism, along with cysteine and methionine metabolism were identified as the potential metabolic pathways that are associated with bovine colostrum milk consumption. In conclusion, consuming bovine colostrum milk may induce metabolic changes and reduce the expression of various pro-inflammatory mediators, thus improving the immune function in older adults.

## 1. Introduction

The aging trend is a global phenomenon, with every region of the world experiencing increases in the proportion of older individuals. According to the United Nations, the global population aged 65 and older has been projected to reach 1.5 billion by 2050, indicating an increase from 9.3% to 16% of the worldwide population aged 65 years and above [1]. This trend is a consequence of declining fertility rates and increasing life expectancies, which have led to a significant shift in the age structure of many societies. The global aging trend represents a major demographic shift with wide-ranging implications for both societies and economies worldwide. One major challenge is its strain on healthcare systems, as older individuals are more likely to require medical attention and long-term care [2]. Additionally, aging populations may pressure social security systems and pension plans, as fewer working-age individuals support retired individuals [3]. As the proportion of older individuals continues to increase, policymakers and individuals alike need to anticipate and address the challenges and opportunities presented by this trend.

Aging and inflammation are strongly associated with each other. As we age, our body undergoes several changes, including a gradual decline in immune function, which results in chronic low-grade inflammation [4]. Inflammation is a natural response of the body’s immune system to injury, infection, or tissue damage. However, this response can become prolonged and excessive in older adults, leading to age-related diseases such as cardiovascular disease, cancer, and neurodegenerative diseases [5]. Inflammation is characterized by the increased production of pro-inflammatory cytokines, reactive oxygen species (ROS), and other immune system molecules [6]. Lifestyle factors such as a poor diet, sedentary behavior, and stress can further exacerbate this chronic inflammation [7]. Thus, targeting inflammation may offer promising interventions to strengthen immune function, prevent age-related diseases, and thus improve the health span.

Colostrum milk, also known as “first milk”, is a highly nutritious and specialized type of milk produced by mammals during the first few days after giving birth [8]. Colostrum milk is packed with essential nutrients, including proteins, carbohydrates, and vitamins, as well as high levels of antibodies and immune factors that help in protecting newborns from infections and illnesses [8,9]. In addition to its immune-boosting properties, colostrum milk has been found to support gut health and improve athletic performance [8,9,10]. Bovine colostrum contains a variety of growth factors that can help support tissue repair and regeneration, making it an attractive supplement for athletes and individuals recovering from injury [8,9,10]. Moreover, bovine colostrum has been shown to exert antioxidant properties due to the presence of antioxidative enzymes (such as glutathione peroxidase, superoxide dismutase, and catalase), low molecular antioxidants and proteins (such as lactoperoxidase, lactoferrin, and ceruloplasmin), vitamins (including vitamins A, C, and E) and minerals (such as selenium and zinc) [11,12]. Supplementation with bovine colostrum has been demonstrated to exert its antioxidative effects in an intestinal ischemia/reperfusion rat model and in the skeletal muscle of mice after performing exercise [13,14]. Since aging is consistently associated with various age-related degenerative diseases due to redox imbalance and a declination in immune function [4,5,15], we postulated that consuming bovine colostrum milk may help to boost the immune system, alleviate the oxidative condition, and support the overall health of older adults, hence promoting successful aging in their late life. Therefore, this double-blind, randomized control trial aimed to investigate the effects of bovine colostrum-enriched skim milk on the expression of various biomarkers on inflammation, antioxidant and oxidative damage, nutrient metabolism, and genomic stability. In addition, metabolomics analysis was also conducted to examine the metabolic changes following the consumption of bovine colostrum-enriched skim milk, thus increasing our understanding of the mechanisms underlying the beneficial effects of consuming bovine colostrum among older adults. 

## 2. Materials and Methods

### 2.1. Ethics Approval and Informed Consent

This study protocol was approved by the Medical Research and Ethics Committee of Universiti Kebangsaan Malaysia (JEP-2021-174) and was conducted in accordance with the Declaration of Helsinki and Good Clinical Practice Guidelines. Written informed consent was obtained from all the participants before data collection.

### 2.2. Study Design and Participants

The study was a 12-week randomized, double-blinded, placebo-controlled trial. The sample size calculation was performed using GPower software version 3.0.10. This study used the mixed model analysis of variance (ANOVA) and repeated measures to analyze the time, group, and interaction effects of the intervention. Therefore, the F-test (ANOVA: repeated measure, within-between interactions) was chosen. The study power was set at 80%, the alpha value at 0.05, and the medium effect size = 0.4, respectively. Based on these parameters, a minimum sample of 52 subjects was required. After considering the 20% dropout rate, a total of 66 participants was required, with 33 subjects in each arm. Older adults aged between 50–69 years were included in this study.

**Figure 1:**
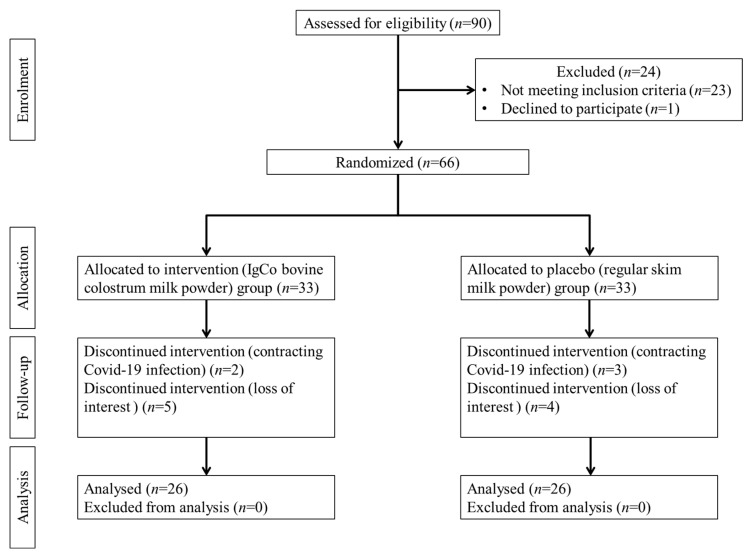
The consort study flow chart. After screening for eligibility, a total of 66 participants who agreed to participate in the study were randomized into the IgCo bovine colostrum supplement group (*n* = 33) and the placebo group (*n* = 33), respectively. There were 14 dropouts, with seven each in both the intervention and placebo groups during follow-up. Hence, only 52 participants (*n* = 52) were included in the analysis, with 26 participants in the intervention and placebo groups, respectively.

Meanwhile, the exclusion criteria for this study were participants who had a current or past history of cancer or were on a chemotherapeutic regimen, allergic/intolerant to dairy products, chronic kidney diseases/kidney failure, uncontrolled hypertension or diabetes, and heart or cardiovascular disease. After screening for eligibility, a total of 66 participants were recruited. The participants were then randomly divided into two groups using computer-generated software based on their gender: the IgCo bovine colostrum supplement group (*n* = 33) and the placebo group (*n* = 33). There were 14 dropouts, with seven each in both the intervention and placebo groups, either due to a loss in interest in following the study or contracting COVID-19 infection during the intervention period, thus being unable to continue with the investigation. Hence, only 52 participants (*n* = 52) were included in the present analysis, with 26 participants in the intervention and placebo groups, respectively. The consort flow chart of the study is shown in Figure 1. Then, ten age- and gender-matched participants were randomly selected from each group and were subjected to metabolomics analysis.

### 2.3. Investigational Product

The investigational product of this trial was IgCo bovine colostrum-enriched skim milk powder, which contains 150 mg of IgG in every sachet (15 g) in the form of pasteurized milk powder. Meanwhile, the placebo for this study was regular skim milk powder (15 g per sachet). The IgCo bovine colostrum and placebo skim milk powder were produced and sponsored by the company SNI SDN BHD. The IgCo bovine colostrum and placebo skim milk were identical in both appearance and taste. Each participant was instructed to consecutively consume the IgCo bovine colostrum or placebo skim milk in two sachets daily for 12 weeks. The nutrition composition of the IgCo bovine colostrum and placebo skim milk is available in the Supplementary Materials. 

### 2.4. Data Collection

The investigators, enumerators, and study participants were blinded from knowing the intervention group allocation throughout the study and during data collection. The participants were interviewed face-to-face by well-trained enumerators to collect the required information, such as sociodemographic factors and self-reported medical history using a standardized questionnaire. Meanwhile, the participants’ height, weight, and circumference of the waist and hip were measured using a SECA 206 portable body meter (Seca, Hamburg, Germany), Tanita digital lithium weighing scale (Tanita, Tokyo, Japan) and Lufkin tape, respectively [16,17]. The body mass index (BMI) of the participants was then calculated by using the formula “body weight (kg)/height (m)^2^. Meanwhile, the systolic and diastolic blood pressure was taken twice using an automatic digital blood pressure monitor (OMRON, Kyoto, Japan) to obtain the average reading.

### 2.5. Blood Samples Collection and Clinical Laboratory Testing

A trained phlebotomist collected fasted venous blood samples from each participant during the baseline and 12th week of intervention. Participants were instructed to fast for at least 8 h prior to blood collection. Following centrifugation, plasma samples were separated from the blood and stored in a −80 °C freezer until further analysis [18,19].

### 2.6. Biomarkers Detection

A total of 8 biomarkers of interest under different categories of biological process were investigated in this study, including antioxidant and oxidative damage [including superoxide dismutase (SOD) activity, malondialdehyde (MDA), and 8-hydroxy-2’-deoxyguanosine (8-OHdG)], inflammation [C-reactive protein (CRP), interleukin 6 (IL-6), and tumor necrosis factor α (TNF-α)], nutrient metabolism [vitamin D], and genomic stability [telomerase]. Commercially available metabolism assay kits that were used for the detection of SOD activity and MDA levels, as well as the ELISA kits that were used for the detection of 8-OHdG, CRP, IL-6, TNF-α, vitamin D, and telomerase, were all purchased from Elabscience Biotechnology Co., Ltd. (Wuhan, China). The metabolism assays and the ELISA tests were conducted based on the protocol stated in the user manual. 

### 2.7. Untargeted Metabolomics Analysis

#### 2.7.1. Samples Preparation

A total of 100 μL of each plasma sample was mixed with 700 μL of extractant containing internal standard (methanol: acetonitrile: water in the ratio of 4:2:1 *v*/*v*/*v*). After shaking for 1 min, the mixtures were placed in a −20 °C refrigerator for 2 h. After centrifugation at 25,000× *g* and 4 °C for 15 min, the supernatants were separated, and 600 μL of each supernatant was transferred into a new microcentrifuge tube. The transferred samples were then dried using a drying machine. The samples were reconstituted by adding 180 μL of methanol: pure water (1:1 *v*/*v*) and mixed for 10 min via vortexing. The samples were centrifuged at 25,000× *g* and 4 °C for 15 min, and the supernatants were then transferred to a new microcentrifuge tube. Lastly, 20 μL of each sample was mixed with the QC samples before proceeding to UPLC-MS analysis.

#### 2.7.2. UPLC-MS Analysis

This experiment used Waters 2777c UPLC (Waters, Mildford, MA, USA) in series with the Q Exactive HF high-resolution mass spectrometer (Thermo Fisher Scientific, Waltham, MA, USA) to separate and detect the metabolites. Briefly, chromatographic separation was performed on a Waters ACQUITY UPLC BEH C18 column (1.7 μm, 2.1 mm × 100 mm; Waters, Mildford, MA, USA), and the column temperature was maintained at 45 °C. The mobile phase composition for the positive mode consisted of 0.1% formic acid (A) and acetonitrile (B); whereas, for the negative mode, it comprised 10 mM ammonium formate (A) and acetonitrile (B). The gradient conditions were as follows: 0–1 min, 2% B; 1–9 min, 2–98% B; 9–12 min, 98% B; 12–12.1 min, 98% B to 2% B; and 12.1–15 min, 2% B, respectively. The flow rate was 0.35 mL/min, and the injection volume was 5 μL. Then, primary and secondary mass spectrometry data acquisition was performed using Q Exactive HF (Thermo Fisher Scientific, USA). The full scan range was 70–1050 m/z with a resolution of 120,000, and the automatic gain control (AGC) target for MS acquisitions was set to 3 × 10^6^ with a maximum ion injection time of 100 ms. The top 3 precursors were selected for subsequent MS/MS fragmentation with a maximum ion injection time of 50 ms and resolution of 30,000, and the AGC was set to 1 × 10^5^, respectively. The stepped normalized collision energy was set to 20, 40, and 60 eV, respectively. The ESI parameters setting was as follows: the sheath gas flow rate was 40, the aux gas flow rate was 10, the positive-ion mode spray voltage(|KV|) was 3.80, the negative-ion mode spray voltage(|KV|) was 3.20, the capillary temperature was 320 °C, and the aux gas heater temperature was 350 °C, respectively.

#### 2.7.3. Metabolite Ion Peak Extraction and Metabolite Identification

After importing the off-line data of mass spectrometry into Compound Discoverer 3.3 (Thermo Fisher Scientific, San Jose, CA, USA) software and analyzing the mass spectrometry data in combination with the BGI metabolome database, mzCloud database, and ChemSpider online database, a data matrix containing information such as metabolite peak area and identification results was obtained.

#### 2.7.4. Bioinformatics Analysis

The result files from the Compound Discoverer were transferred to R software package metaX (BGI Shenzhen, Guangdong, China) for data pre-processing and further analysis. During pre-processing, the data were normalized to obtain the relative peak areas by probabilistic quotient normalization (PQN). Then, the batch effects were corrected using quality control-based robust LOESS signal correction. Metabolites with a coefficient of variation larger than 30% on their relative peak area in QC samples were then removed from further analysis. Subsequently, multivariate statistical and univariate analyzes were used to screen different metabolites between the groups, with slight modifications from the previously described methods [20]. The pre-processed data was log-transformed and auto-scaled in the Pareto scale. Then, principal component analysis (PCA) was performed to reflect the actual distribution of samples and to observe the separation trend between the sample groups. Partial least squares-discriminant analysis (PLS-DA), a supervised statistical method used to enhance the differentiation between the classification groups, was subsequently conducted. The PLS-DA model was established between the comparative analysis groups (two groups of samples), and a 5-fold cross-validation was used to validate when building the model. Then, the orthogonal partial least squares discriminant analysis (OPLS-DA) was performed on two groups of biological samples. The purpose was to establish the relationship model between the metabolite expression and sample categories, thereby allowing for the modelling and prediction of these sample categories. At the same time, the ability of each metabolite to classify and distinguish each group of samples was measured by calculating the variable important for the projection (VIP). For the screening of metabolic biomarkers, it is generally considered that a VIP greater than 1 indicates that the variable significantly affects the differentiation of the sample categories.

For univariate analysis of the data, the differences in metabolite concentration were evaluated in terms of fold change, and statistical comparison was conducted using the independent *t*-test. The displayed metabolites with a *p*-value < 0.05 indicated significant differences in the fold-change values. Only the statistically significant metabolites were considered as differential metabolites. Metabolites with a VIP value ≥ 1, fold change ≥ 1.2 or ≤0.8, or *p*-value < 0.05 were considered as differential metabolites. 

After the differential metabolites were screened, the expression patterns of the differential metabolites were analyzed by clustering analysis, correlation clustering, and network analysis. Additionally, biological functions were explored through pathway annotation and pathway enrichment analysis. Using the Euclidean distance method, Hierarchical cluster analysis (HCA) was applied to examine the expression levels of the differential metabolites in the two different groups. The identified metabolites’ taxonomic and functional annotations were performed by comparing the KEGG and HMDB databases. Afterward, enrichment and pathway analyzes were conducted using the MetaboAnalyst 5.0 software. 

### 2.8. Statistical Analysis

Statistical Package for Social Science (SPSS) version 26 software was used to conduct all statistical analyzes at a significance level of *p* < 0.05. Descriptive analysis was performed using the independent *t*-test and chi-square test for continuous and categorical data, respectively. Then, the paired sample *t*-test was employed to examine the effects of the intervention on the expression levels of various biomarkers within the same treatment group by comparing the measurements before and after the treatment. Lastly, a two-way repeated measure ANOVA analysis was used to study the effects of IgCo bovine colostrum milk supplementation on biomarker outcomes, specifically the impact of time, group, and interactions between them. After the Bonferroni correction, the model was adjusted for various potential confounding factors including age and sex. 

## 3. Results

### 3.1. The Baseline Attributes of Participants

Table 1 shows the baseline attributes of the participants. The mean age of all the participants was 61.71 ± 7.14, with the majority of the participants being female (55.8%), married (96.2%), non-smokers (96.2%), having BMI of 27.19 ± 4.61 kg/m^2^, waist circumference of 90.32 ± 10.20 cm, hip circumference of 102.60 ± 8.80 cm, systolic blood pressure of 133.78 ± 16.13 mmHg, and diastolic blood pressure of 82.61 ± 9.79 mmHg, respectively. None of the aforementioned parameters showed significant differences across the intervention and placebo groups (*p* > 0.05). Regarding medical history, 21.2% of participants had diabetes, 28.8% had hypertension, and 32.7% had hypercholesterolemia, respectively. The distribution of comorbidities was found to be non-significant across the groups.

### 3.2. The Effects of 12 Weeks Intervention on the Expression of Biomarkers

Table 2 shows the effects of 12 weeks IgCo bovine colostrum milk supplementation on the levels of various biomarkers among the participants. Our current findings show that following 12 weeks of intervention, there were significant interaction effects observed between the group and time (*p* < 0.05) in the expression of various inflammatory biomarkers, namely the CRP, IL-6, and TNF-α. Specifically, there was a significant reduction (*p* < 0.05) observed in the expression levels of CRP (5.03 ± 3.26 to 3.34 ± 2.00 ng/mL), IL-6 (3.32 ± 1.19 to 2.67 ± 1.13 pg/mL), and TNF-α (91.88 ± 56.80 to 51.31 ± 44.07 pg/mL) among participants who consumed the IgCo bovine colostrum milk. It is noted that participants in the placebo group also showed a significant reduction in TNF-α expression levels (80.26 ± 46.85 to 64.51 ± 37.03 pg/mL, respectively) after the intervention. However, no significant interaction effects of the group and time were observed in the levels of vitamin D, telomerase, and antioxidant and oxidative damage biomarkers (8-OHdG, MDA, and SOD activities).

### 3.3. Metabolic Profiles of Bovine Colostrum Supplemented Group and Placebo Group

**Figure 2:**
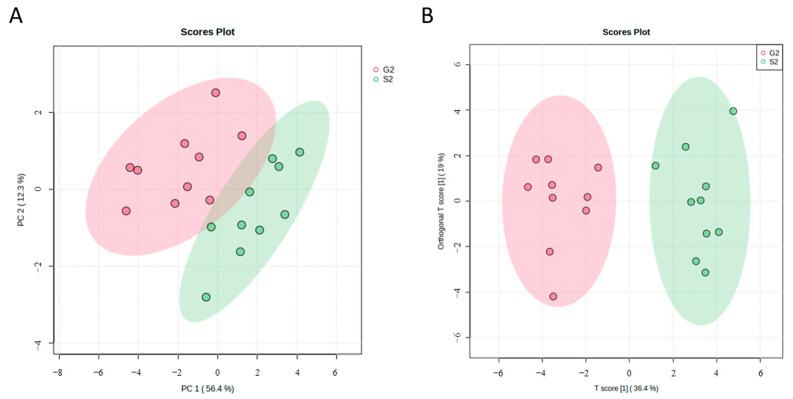
(**A**) PCA and (**B**) OPLS-DA score plot of the placebo group (red color) and the treatment group (green color) in plasma samples after 12 weeks of intervention. For clarity, the ellipses show the 95% confidence region.

To determine the metabolic changes following the consumption of IgCo colostrum milk, untargeted metabolomics analysis was conducted using the UPLC-MS approach. The serum profiles of the IgCo supplemented (G2) and placebo (S2) groups were analyzed using the multivariate data analyzes approach to provide a global view of the metabolic alterations. PCA was used to perform unsupervised multivariate analysis of the serum groups. The results showed an apparent separation between the IgCo-supplemented and placebo groups on the scores plot of PCA (Figure 2A). All the samples in each group were located in a 95% confidence interval. Based on findings from the OPLS-DA analysis, the first predictive component T score[1] (x-axis) explained 36.4% of the variation between the groups, while the orthogonal T score[1] (y-axis) accounted for 19.0% of the variation within the groups. The OPLS-DA model encompasses a good internal cumulative cross-validation of the goodness of fit and a good predictive ability (R2Y = 0.818, Q2 = 0.782). The discrete clusters by PC1 are shown in Figure 2B. From the OPLS-DA score plot, it can be observed that the IgCo supplemented (G2) and placebo (S2) groups were clearly distinguishable from each other by the first predictive component T score[1] (x-axis).

### 3.4. Detection and Identification of Metabolic Markers

A total of 33 differential metabolites between the intervention and placebo groups were identified, with 22 metabolites upregulated (2,4,12-Octadecatrienoic acid isobutylamide, 4,4’-diapolycopenedial, docosanamide, ethyl acetate, gamma-glutamylglutamic acid, gamma-linolenic acid, glycerophosphocholine, indolepyruvate, isovaleric acid, leukotriene C5, leukotriene E3, linoleamide, LysoPC(16:1(9Z)), LysoPC(18:3(9Z,12Z,15Z)), LysoPE(18:0/0:0), N,N-dimethylsphingosine, N-arachidonoyl dopamine, n-butyl acetate, N-oleoylethanolamine, octadecanamide, oleamide and propionic acid) and 11 metabolites were downregulated (3-hydroxybutyric acid, 3-oxododecanoic acid, But-2-enoic acid, etiocholanolone, PC(o-18:1(9Z)/16:0), PE(O-18:1(1Z)/20:4(5Z,8Z,11Z,14Z)), PE(P-16:0/20:3(8Z,11Z,14Z)), PE(P-18:0/18:2(9Z,12Z)), PE(P-18:0/22:6(4Z,7Z,10Z,13Z,16Z,19Z)), PE(P-18:1(9Z)/22:6(4Z,7Z,10Z,13Z,16Z,19Z)), and ubiquinone-4) in the intervention group compared to the placebo group. The fold change, state, *p*-value, and VIP value of each differential metabolite are depicted in Table 3. The variation of these identified differential metabolites among the two groups was extended through the HCA analysis, as shown in Figure S1.

Furthermore, to determine the within-group differences in the metabolic markers, the differential metabolites within the IgCo bovine colostrum milk-supplemented and placebo groups pre- and post-intervention were also determined, as shown in Tables S2 and S3, respectively. Overall, 48 (21 upregulated and 27 downregulated) and 37 (16 upregulated and 21 downregulated) differential metabolites were identified in the IgCo bovine colostrum milk-supplemented and placebo groups pre- and post-intervention, respectively.

### 3.5. Characterization and Functional Analysis of Metabolic Pathways

**Figure 3:**
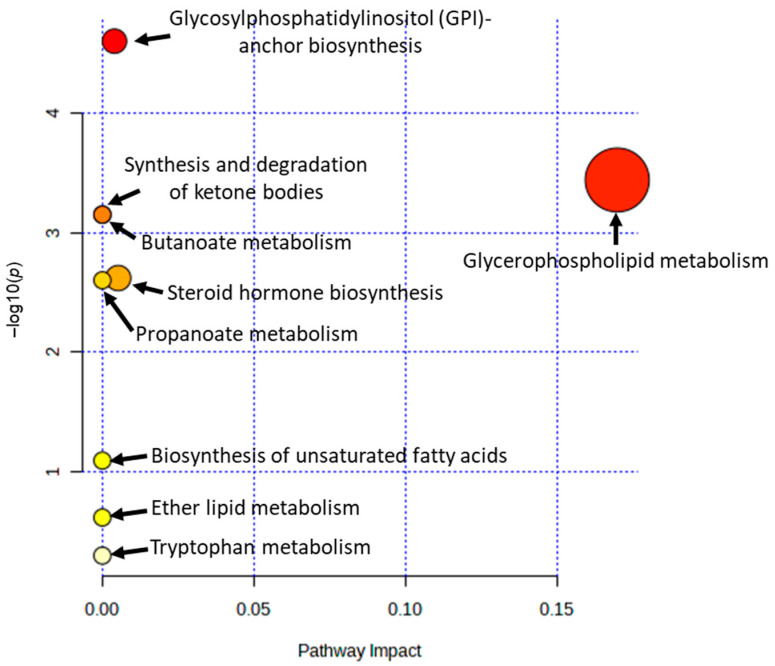
The summary of pathway analysis with the MetPA system (MetaboAnalyst 5.0). The metabolic pathways were represented as circles according to their –log(*p*) value at the y-axis and their pathway impact at the x-axis. The darkness and size of each circle corresponds to the –log(*p*) value and impact score of the metabolic pathway, respectively.

Metabolic pathways were defined with an online MetPa system (MetaboAnalyst 5.0). Nine suggested metabolic pathways were found to be associated with the consumption of IgCo bovine colostrum milk compared to the regular skim milk in the placebo group. The potential metabolic pathway was identified based on impact value > 0.1 and a *p*-value less than 0.05. Out of the nine suggested metabolic pathways, only the glycerophospholipid metabolism pathway fulfilled the criteria mentioned above and was determined as the potential metabolic pathway in this model. Table 4 shows the output of pathway analysis from the MetPa system, and the findings are summarized in Figure 3. Meanwhile, the potential metabolic pathway before and after consuming the IgCo bovine colostrum milk and regular skim milk were depicted in Tables S4 and S5, respectively. The possible metabolic pathways associated with bovine colostrum milk consumption were glycerophospholipid metabolism and cysteine and methionine metabolism. Meanwhile, sphingolipid and glycerolipid metabolism were found to be the potential metabolic pathways related to regular skim milk consumption.

## 4. Discussion

This study aimed to investigate the effects of bovine colostrum-enriched skim milk on the expression of various biomarkers on inflammation, antioxidant and oxidative damage, nutrient metabolism, and genomic stability. Our current findings show a significant reduction in the expression of various pro-inflammatory mediators, such as the CRP, IL-6, and TNF-α following the consumption of IgCo bovine colostrum milk, suggesting that it can help in the regulation of immune function and improve the inflammatory conditions in the body. However, consumption of bovine colostrum milk did not have any significant interaction effects of the group and time in relation to the levels of vitamin D, telomerase, 8-OHdG, MDA, and SOD activity. Moreover, the untargeted metabolomics analysis using the UPLC-MS approach revealed that consuming bovine colostrum milk may be associated with alterations in glycerophospholipid metabolism and cysteine and methionine metabolism.

Bovine colostrum is a rich source of bioactive molecules, including immunoglobulins, growth factors, cytokines, and peptides that have anti-inflammatory properties [8,9,10]. The anti-inflammatory effects of bovine colostrum have been attributed to several mechanisms, including the modulation of immune responses, inhibition of pro-inflammatory cytokines and enzymes, and promotion of tissue repair [8,9,21]. One of the key bioactive molecules in bovine colostrum is lactoferrin, which has been shown to have anti-inflammatory effects [21,22]. Lactoferrin can bind to bacterial and viral cell walls, preventing them from attaching to host cells and reducing the production of pro-inflammatory cytokines [22]. In addition, lactoferrin can modulate the immune system by increasing the number and activity of natural killer cells, which play a crucial role in eliminating the infected cells [23]. Bovine colostrum also contains a variety of growth factors, including insulin-like growth factor 1 (IGF-1), transforming growth factor beta (TGF-β), and epidermal growth factor (EGF) [8]. These growth factors can promote tissue repair and regeneration, which is essential in resolving inflammation.

Furthermore, bovine colostrum contains peptides that have been shown to have anti-inflammatory properties. One of these peptides is lactoperoxidase, which has been shown to inhibit the pro-inflammatory hydrogen peroxide-induced IL-8 secretion in Caco-2 cells [24]. Deleting the lactoperoxidase gene has been demonstrated to cause inflammation in various organs of mice [25]. Meanwhile, another peptide known as lactoferrin has been shown to have an immunomodulatory function and exert anti-inflammatory properties by engaging with diverse cell receptors and activating various cell signaling pathways, frequently facilitated by iron-dependent mechanisms [26]. Moreover, human chondrocytes treated with lactoferrin from exogenous sources have been proven to inhibit the activation of the NF-κB signal transduction pathway, cyclooxygenase-2 expression, and prostaglandin E2 production following IL-1β-stimulation [27]. Previously, orally administered colostrum polyvalent immunoglobulins have been demonstrated to decrease the secretion of IL-1β, IL-6, interferon (IFN)-γ, TNF-α, and IL-12 inflammatory cytokines in patient-derived peripheral blood mononuclear cells (PBMCs) [28]. Additionally, the supplementation of bovine colostrum concentrate among male endurance athletes have been found to inhibit the secretion of TNF, IL-6, and IL-4 from PBMCs in the early phase after adding lipopolysaccharide (LPS) [29]. Hence, suppressing the expression of pro-inflammatory cytokines after consuming bovine colostrum milk may improve immune function.

Glycerophospholipids are a type of phospholipid that form the structural basis of cell membranes [30]. Previous studies have suggested that glycerophospholipid metabolism is implicated in overall systemic immune function and low-grade inflammation, with phospholipids potentially acting as mediators of inflammation [31,32]. Glycerophospholipids play a crucial role in regulating inflammation by serving as precursors for a wide range of signaling molecules, including prostaglandins, leukotrienes, and platelet-activating factors (PAF) [33,34]. These lipid mediators are synthesized by a series of enzymatic reactions that are tightly regulated in response to cellular signals. Research has shown that alterations in the composition of glycerophospholipids can profoundly affect the inflammatory response. For example, studies have indicated that supplementation with glycerophospholipid phosphatidylcholine (PC) can decrease the production of inflammatory cytokines in response to LPS, which is a potent activator of the immune system [35].

In addition, glycerophospholipids have been found to play a role in inflammation by modulating the activity of immune cells and regulating the production of inflammatory mediators. Glycerophospholipids may contribute to inflammation by interacting with toll-like receptors (TLR), thus inhibiting the activation of TLR2 and TLR4 induced by pathogen-associated molecular patterns [36]. In addition, studies have proven that phospholipids, such as PC and phosphatidylethanolamine (PE), can be subjected to hydrolysis by the enzyme phospholipase A2 (PLA2) to release arachidonic acid from cell membranes [37]. Arachidonic acid is a precursor to various inflammatory mediators, including prostaglandins and leukotrienes [38]. Furthermore, the dysregulation in the glycerophospholipid metabolism pathway has been shown to affect the inflammatory response and has been implicated in the development of various diseases associated with chronic inflammation, such as Behçet’s disease, allergic airway disease, and rheumatoid arthritis [39,40,41]. Interestingly, a previous study found that most PCs or Pes were negatively correlated with the inflammatory index, particularly those containing more polyunsaturated acyl chains [42]. Conversely, those with lower levels of polyunsaturated acyl chains were more likely to correlate positively with inflammation. As most of the identified PCs and PEs in this present study were those with lower levels of polyunsaturated acyl chains, the reduction in the expression of the pro-inflammatory biomarkers may be due to the alterations in such PC and PE metabolites following the consumption of the IgCo bovine colostrum milk.

In addition to the glycerophospholipid metabolism pathway, our current findings also demonstrate that cysteine and methionine metabolism is one of the potential metabolic pathways associated with bovine colostrum milk consumption pre- and post-intervention. Following the intervention, a decrease in the expression of methionine, one of the metabolites involved in the cysteine and methionine metabolism, was observed. Methionine is an essential amino acid that plays a vital role in protein synthesis and many other metabolic processes including immunity [43,44]. It has also been linked to inflammation, with several studies suggesting that high levels of methionine intake may contribute to chronic inflammation. One way methionine may contribute to inflammation is through the generation of homocysteine, a toxic amino acid that can damage cells and tissues [45]. Elevated homocysteine levels have been found to be associated with increased inflammation, oxidative stress, and a higher risk of cardiovascular disease [45,46,47]. Furthermore, methionine metabolism can also lead to the production of S-adenosylmethionine (SAMe), a methyl donor involved in various cellular processes, including the regulation of gene expression. Studies have shown that alterations in the SAMe levels can affect the activation of the mitogen-activated protein kinase (MAPK) signaling pathway, thus reducing the production of TNF-α, IL-6, and interferon-β, respectively [48]. Moreover, a previous study demonstrated that consuming a methionine restriction diet can mitigate the inflammation associated with aging and obesity in the liver, visceral, and subcutaneous white adipose tissue [49]. Thus, the reduction in the expression levels of these pro-inflammatory mediators may be due to the decrease in the methionine levels following the consumption of IgCo bovine colostrum milk.

## 5. Conclusions

In conclusion, consuming IgCo bovine colostrum-enriched skim milk may help reduce the expression levels of various pro-inflammatory mediators, such as the CRP, IL-6, and TNF-α. Findings from the untargeted metabolomics analysis revealed that consuming bovine colostrum may induce alterations in the glycerophospholipid metabolism and cysteine and methionine metabolism pathways, thus improving the immune function in older adults. Our current findings suggest that bovine colostrum milk has the potential to be used as one of the nutraceutical foods in promoting healthy and successful aging.

## Figures and Tables

**Table 1:** The baseline attributes of participants, total and by intervention group.

Parameters	*n* (%) or Mean ± SD			
	Total 52 (100.0)	Bovine Colostrum Milk 26 (50.0)	Placebo 26 (50.0)	*p*-Value
Age	61.71 ± 7.14	60.46 ± 7.07	62.96 ± 7.13	0.210
Sex				
Male	23 (44.2)	11 (42.3)	12 (46.2)	0.780
Female	29 (55.8)	15 (57.7)	14 (53.8)	
Marital status				
Single/Divorced	2 (3.8)	0 (0.0)	2 (7.7)	0.149
Married	50 (96.2)	26 (100.0)	24 (92.3)	
Smoking status	2 (3.8)	1 (3.8)	1 (3.8)	1.000
BMI (kg/m^2^)	27.19 ± 4.61	26.55 ± 5.16	27.84 ± 3.99	0.318
Waist circumference (cm)	90.32 ± 10.20	88.03 ± 11.15	92.81 ± 8.60	0.098
Hip circumference (cm)	102.60 ± 8.80	100.49 ± 8.62	104.89 ± 8.58	0.077
Systolic pressure (mmHg)	133.78 ± 16.13	130.65 ± 13.94	136.90 ± 17.79	0.165
Diastolic pressure (mmHg)	82.61 ± 9.79	81.37 ± 10.14	83.85 ± 9.45	0.366
Medical history				
Diabetes	11 (21.2)	4 (15.4)	7 (26.9)	0.308
Hypercholesterolemia	17 (32.7)	9 (34.6)	8 (30.8)	0.768
Hypertension	15 (28.8)	7 (26.9)	8 (30.8)	0.760

Note: Data were presented as mean ± SD or *n* (%). Descriptive analysis was performed using the independent *t*-test and chi-square test for continuous and categorical data, respectively. No significant differences were detected between the intervention and placebo groups (*p* > 0.05) in all the baseline attributes.

**Table 2:** The effects of 12 weeks of IgCo bovine colostrum milk supplementation on biomarkers among participants.

	Bovine Colostrum Milk (*n* = 26)	Placebo (*n* = 26)	Group Effect			Time Effect			Group × Time Effect		
			*p*	Partial Eta Squared	Power	*p*	Partial Eta Squared	Power	*p*	Partial Eta Squared	Power
Vitamin D (µmol/L)											
Baseline	105.74 ± 53.61	142.22 ± 60.79	0.012 *	0.125	0.729	0.265	0.026	0.197	0.119	0.050	0.343
Post	60.64 ± 24.05 ^###^	70.28 ± 50.52 ^###^									
MDA (µmol/L)											
Baseline	0.74 ± 0.40	1.14 ± 0.91	0.047 *	0.080	0.515	0.586	0.006	0.084	0.490	0.010	0.105
Post	1.11 ± 0.48 ^##^	1.31 ± 0.75									
SOD activity (U/mL)											
Baseline	122.23 ± 12.42	122.88 ± 25.13	0.306	0.022	0.173	0.002	0.175	0.879	0.942	0.000	0.051
Post	113.12 ± 11.36 ^##^	117.04 ± 20.47									
8-OHdG (ng/mL)											
Baseline	37.16 ± 36.43	30.03 ± 15.18	0.842	0.001	0.054	0.163	0.040	0.285	0.196	0.035	0.251
Post	42.76 ± 38.95	45.83 ± 44.50									
CRP (ng/mL)											
Baseline	5.03 ± 3.26	4.70 ± 1.91	0.640	0.005	0.075	0.657	0.004	0.072	0.015 *	0.117	0.694
Post	3.34 ± 2.00 ^##^	4.55 ± 2.16									
IL-6 (pg/mL)											
Baseline	3.32 ± 1.19	3.45 ± 1.43	0.082	0.062	0.413	0.156	0.041	0.292	0.018 *	0.111	0.671
Post	2.67 ± 1.13 ^#^	3.64 ± 1.49									
TNF-α (pg/mL)											
Baseline	91.88 ± 56.80	80.26 ± 46.85	0.898	0.000	0.052	0.717	0.003	0.065	0.008 **	0.138	0.774
Post	51.31 ± 44.07 ^###^	64.51 ± 37.03 ^#^									
Telomerase (ng/mL)											
Baseline	456.29 ± 179.56	465.68 ± 193.06	0.915	0.000	0.051	0.070	0.067	0.443	0.819	0.001	0.056
Post	495.59 ± 185.98	500.60 ± 229.12									

Note: The two-way repeated measured ANOVA model was adjusted with confounding factors such as age and gender. Abbreviation: 8-OHdG, 8-hydroxy-2-deoxyguanosine; CRP, C-reactive protein; IL-6, interleukin 6; MDA, malondialdehyde; SOD, superoxide dismutase; and TNF- α, tumor necrosis factor α. * *p* < 0.05; ** *p* < 0.01, significant different in two-way repeated measured ANOVA. ^#^ *p* < 0.05; ^##^ *p* < 0.01; ^###^ *p* < 0.001, significant differences before and after the intervention within the same treatment group.

**Table 3:** Relative quantification of the major serum metabolites between the intervention and placebo groups post-intervention.

Metabolite	Fold Change	State	*p*-Value	VIP
Propionic acid	18.634	Up	0.001	5.251
Isovaleric acid	9.308	Up	0.003	4.594
n-butyl acetate	31.712	Up	0.004	4.464
Ethyl acetate	43.492	Up	0.016	4.306
Gamma-glutamylglutamic acid	10.617	Up	0.010	3.876
Linoleamide	2.749	Up	0.000	3.250
4,4’-diapolycopenedial	2.678	Up	0.000	3.012
Oleamide	2.247	Up	0.000	2.882
2,4,12-octadecatrienoic acid isobutylamide	2.563	Up	0.000	2.702
Leukotriene E3	2.268	Up	0.000	2.511
Gamma-linolenic acid	1.937	Up	0.001	2.240
N-oleoylethanolamine	2.049	Up	0.017	2.121
Etiocholanolone	0.467	Down	0.037	1.934
Glycerophosphocholine	1.832	Up	0.002	1.913
Octadecanamide	1.733	Up	0.000	1.911
N,N-dimethylsphingosine	1.747	Up	0.007	1.807
N-arachidonoyl dopamine	1.755	Up	0.004	1.775
Leukotriene C5	1.668	Up	0.013	1.759
3-oxododecanoic acid	0.689	Down	0.036	1.700
PE(P-18:0/18:2(9Z,12Z))	0.480	Down	0.018	1.605
PE(O-18:1(1Z)/20:4(5Z,8Z,11Z,14Z))	0.629	Down	0.014	1.552
But-2-enoic acid	0.563	Down	0.046	1.479
3-hydroxybutyric acid	0.694	Down	0.035	1.462
PE(P-18:0/22:6(4Z,7Z,10Z,13Z,16Z,19Z))	0.652	Down	0.003	1.407
LysoPC(18:3(9Z,12Z,15Z))	1.319	Up	0.004	1.405
LysoPE(18:0/0:0)	1.350	Up	0.006	1.368
Ubiquinone-4	0.725	Down	0.023	1.266
PC(o-18:1(9Z)/16:0)	0.673	Down	0.005	1.212
LysoPC(16:1(9Z))	1.383	Up	0.040	1.161
PE(P-16:0/20:3(8Z,11Z,14Z))	0.663	Down	0.028	1.141
PE(P-18:1(9Z)/22:6(4Z,7Z,10Z,13Z,16Z,19Z))	0.713	Down	0.039	1.111
Indolepyruvate	1.500	Up	0.038	1.071
Docosanamide	1.396	Up	0.002	1.055

Note: The differences in metabolite concentration were evaluated in terms of fold change, and statistical comparison was conducted using the *t*-test. The displayed metabolites with a *p*-value < 0.05 indicate significant differences in fold-change values between the groups. Only metabolites with VIP value ≥ 1, fold change ≥ 1.2 or ≤0.8, or *p*-value < 0.05 were considered differential metabolites. Up: a relatively higher concentration compared to the placebo group. Down: a relatively lower concentration compared to the placebo group.

**Table 4:** Pathway analysis results (IgCo bovine colostrum milk vs. placebo post-intervention) with the MetPA system (MetaboAnalyst 5.0).

Pathway Name	Hits	Raw *p*	–log(*p*)	Holm Adjust	FDR	Impact
Glycerophospholipid metabolism	3	0.0004	3.4391	0.0029	0.0016	0.1700
Steroid hormone biosynthesis	1	0.0024	2.6193	0.0120	0.0038	0.0052
Glycosylphosphatidylinositol (GPI)-anchor biosynthesis	1	2.52 × 10^−5^	4.5993	0.0002	0.0002	0.0040
Synthesis and degradation of ketone bodies	1	0.0007	3.1494	0.0050	0.0016	0.0000
Butanoate metabolism	1	0.0007	3.1494	0.0050	0.0016	0.0000
Propanoate metabolism	1	0.0025	2.6011	0.0120	0.0038	0.0000
Biosynthesis of unsaturated fatty acids	1	0.0806	1.0936	0.2418	0.1037	0.0000
Ether lipid metabolism	1	0.2411	0.6177	0.4823	0.2713	0.0000
Tryptophan metabolism	1	0.5021	0.2992	0.5021	0.5021	0.0000

Note: The potential metabolic pathway was identified based on an impact value > 0.1 and a *p*-value less than 0.05.

## Data Availability

The data presented in this study are available from the corresponding author on reasonable request.

## Supplementary Materials

The following supporting information can be downloaded at: https://www.mdpi.com/article/10.3390/nu15143184/s1, Figure S1: Heat map of the identified plasma metabolites of individuals in IgCo supplemented group and placebo group after 12 weeks of intervention; Table S1: Nutritional information of IgCo bovine colostrum milk and placebo (regular skim milk); Table S2: Relative quantification of the major serum metabolites within the IgCo supplemented group pre- and post-intervention; Table S3: Relative quantification of the major serum metabolites within the placebo group pre- and post-intervention; Table S4: Results of pathway analysis (IgCo colostrum milk pre- vs post-intervention) with MetPA system (MetaboAnalyst 5.0); Table S5: Results of pathway analysis (Placebo pre- vs. post-intervention) with MetPA system (MetaboAnalyst 5.0).
