# Supplementary material for: The Effects of 12 Weeks Colostrum Milk Supplementation on the Expression Levels of Pro-Inflammatory Mediators and Metabolic Changes among Older Adults: Findings from the Biomarkers and Untargeted Metabolomic Analysis
Source: Nutrients. 2023 Jul 18;15(14):3184. doi: 10.3390/nu15143184 (PMC10384749; doi:10.3390/nu15143184)
Supplement: Supplementary file 1 [file nutrients-15-03184-s001.zip › nutrients-2407043-supplementary.pdf]

Supplementary materials

Table S1. Nutritional information of IgCo bovine colostrum-enriched skim milk and placebo (regular skim milk)

Nutrition	IgCo bovine colostrum-enriched skim milk (per 100g)	Placebo (per 100g)
Energy (kcal)	357	360
Energy/calorie from fat (kcal)	8	10
Fat (g)	0.92	1.11
Carbohydrate (g)	50.4	54.1
Total sugars (g)	38.3	35.6
Protein (g)	36.7	33.5
Zinc (mg)	2.94	3.26
Magnesium (mg)	NA	122
Iron (mg)	ND (<0.10)	0.18
Copper (mg)	ND (<0.10)	NA
Calcium (mg)	5752	1247
Vitamin A 0.241(μg)	ND (<45)	ND (<45)
Vitamin C (mg)	310	ND (<0.10)
Vitamin D3 (μg)	ND (<2)	2.55
Vitamin E (IU)	0.03	0.03
Vitamin B1 (mg)	0.241	0.265
Vitamin B2 (mg)	1.08	4.18
Vitamin B3 (mg)	0.92	NA
Vitamin B5 (mg)	4.11	NA
Vitamin B6 (mg)	0.053	ND (<0.005)
Biotin (μg)	18.1	NA
Folic acid (μg)	2.22	6.08
Vitamin B12 (μg)	0.83	NA
Vitamin K1 (μg)	9.7	NA

Note: NA, data not available; ND, not detected.

Table S2. Relative quantification of the major serum metabolites within the IgCo supplemented group pre- and post-intervention.

Metabolite	Fold Change	State	pvalue	VIP
N-Acetylserotonin	0.057	Down	0.000	5.698
8-Hydroxyalanylclavam	4.725	Up	0.000	3.836
Merodesmosine	2.439	Up	0.002	3.628
3,3',4'5-Tetrahydroxystilbene	0.170	Down	0.000	3.487
9,12,13-TriHOME	0.256	Down	0.000	3.352
6-Keto-prostaglandin F1a	0.317	Down	0.000	3.265
15-Keto-prostaglandin F2a	0.338	Down	0.000	3.264
3,4-Dihydroxymandelic acid	0.270	Down	0.000	3.006
(13E)-11a-Hydroxy-9,15-dioxoprost-13-enoic acid	0.386	Down	0.000	2.828
L-Methionine	0.441	Down	0.006	2.661
Linoleamide	2.213	Up	0.000	2.621
Oleamide	1.908	Up	0.000	2.356
2,4,12-Octadecatrienoic acid isobutylamide	2.133	Up	0.001	2.263
PE(P-16:0e/0:0)	1.777	Up	0.001	2.191
Dolichol phosphate	3.285	Up	0.043	2.190
Deoxycholic acid glycine conjugate	0.512	Down	0.020	2.054
N-Oleoylethanolamine	1.878	Up	0.014	2.028
4,4'-Diapolycopenedial	1.810	Up	0.003	2.004
Monoethylhexyl phthalic acid	2.164	Up	0.031	1.843
PE(P-16:0/18:1(9Z))	0.421	Down	0.015	1.779
PE(P-18:0/18:2(9Z,12Z))	0.474	Down	0.017	1.739
N-arachidonoyl dopamine	1.724	Up	0.008	1.703
Gamma-Linolenic acid	1.641	Up	0.010	1.628
Leukotriene E3	1.680	Up	0.001	1.606
20-Hydroxyeicosatetraenoic acid	1.550	Up	0.015	1.584
SM(d18:0/16:0)	0.440	Down	0.016	1.567
Propionylcarnitine	0.673	Down	0.014	1.544
N,N-Dimethylsphingosine	1.597	Up	0.018	1.535
3-Hydroxyisovaleric acid	0.729	Down	0.034	1.511
Hippuric acid	0.594	Down	0.023	1.477
Sphingosine	1.658	Up	0.008	1.453
SM(d18:1/18:0)	0.550	Down	0.011	1.444
L-Arginine	0.668	Down	0.015	1.409
4-Hydroxyphenylpyruvic acid	0.687	Down	0.005	1.403
L-Acetylcarnitine	0.656	Down	0.008	1.389
PE(20:0/20:2(11Z,14Z))	0.614	Down	0.027	1.334
PE(18:0/22:5(4Z,7Z,10Z,13Z,16Z))	0.723	Down	0.020	1.309
PS(18:0/18:1(9Z))	2.180	Up	0.027	1.241
Iodine	0.755	Down	0.026	1.234
Glycerophosphocholine	1.396	Up	0.017	1.231
2-Methylcitric acid	0.772	Down	0.008	1.210
Octadecanamide	1.406	Up	0.001	1.206
PE(P-16:0/18:2(9Z,12Z))	0.626	Down	0.038	1.206
Sphinganine 1-phosphate	1.300	Up	0.049	1.202

PE(16:0/22:1(13Z))	0.693	Down	0.027	1.163
Methyl stearate	1.467	Up	0.024	1.091
Phosphonoacetaldehyde	0.781	Down	0.040	1.090
Cholesterol sulfate	0.693	Down	0.005	1.005

Table S3. Relative quantification of the major serum metabolites within the placebo group pre- and post-intervention.

Metabolite	Fold Change	State	pvalue	VIP
N-Acetylserotonin	0.160	Down	0.000	5.278
9,12,13-TriHOME	0.215	Down	0.000	4.516
8-Hydroxyalanylclavam	4.365	Up	0.000	4.447
3,3',4'5-Tetrahydroxystilbene	0.198	Down	0.000	3.770
UDP-L-Ara4O	0.298	Down	0.029	3.491
5'-(3',4'-Dihydroxyphenyl)-gamma-valerolactone sulfate	0.310	Down	0.005	3.317
Hypoxanthine	0.521	Down	0.006	3.198
(13E)-11a-Hydroxy-9,15-dioxoprost-13-enoic acid	0.425	Down	0.000	3.014
6-Keto-prostaglandin F1a	0.426	Down	0.000	2.920
Merodesmosine	3.482	Up	0.004	2.871
3,4-Dihydroxymandelic acid	0.343	Down	0.002	2.779
L-Arginine	0.560	Down	0.001	2.475
Thiomorpholine 3-carboxylate	1.961	Up	0.019	2.464
15-Keto-prostaglandin F2a	0.496	Down	0.001	2.463
Sphinganine 1-phosphate	1.685	Up	0.000	2.299
20-Hydroxyeicosatetraenoic acid	1.809	Up	0.001	2.250
Spermidine	0.523	Down	0.016	2.172
But-2-enoic acid	1.741	Up	0.022	1.819
PE(P-18:0/22:4(7Z,10Z,13Z,16Z))	0.611	Down	0.036	1.805
Glycerol	1.569	Up	0.030	1.717
4-Oxoproline	1.294	Up	0.000	1.629
Pyroglutamic acid	1.297	Up	0.000	1.610
Iodine	0.742	Down	0.012	1.530
PE-NMe2(18:1(9Z)/18:1(9Z))	0.688	Down	0.039	1.504
Indolepyruvate	0.621	Down	0.019	1.369
4,4'-Diapolycopenedial	0.673	Down	0.004	1.345
N-Acetyl-L-methionine	0.720	Down	0.028	1.341
Sphinganine	1.522	Up	0.002	1.339
PS(18:0/18:1(9Z))	2.234	Up	0.047	1.326
Alpha-Linolenoyl ethanolamide	1.344	Up	0.014	1.302
Sphingosine	1.520	Up	0.039	1.218
PE(P-16:0/18:1(9Z))	0.673	Down	0.023	1.212
Sphingosine 1-phosphate	1.240	Up	0.006	1.188
LysoPE(18:0/0:0)	0.802	Down	0.021	1.182
N-Acetylneuraminate	0.560	Down	0.028	1.164
Allantoic acid	1.210	Up	0.028	1.154
Dihydrofolic acid	1.557	Up	0.042	1.028

Table S4. Results of pathway analysis (IgCo colostrum milk pre- vs post-intervention) with MetPA system (MetaboAnalyst 5.0).

Pathway name	Hits	Raw p	-log(p)	Holm adjust	FDR	Impact
Ubiquinone and other terpenoid-quinone biosynthesis	1	0.2543	0.5947	1.0000	0.2702	1.0000
Glycerophospholipid metabolism	3	0.0004	3.3491	0.0063	0.0019	0.1996
Cysteine and methionine metabolism	1	0.0150	1.8232	0.1202	0.0255	0.1045
Tyrosine metabolism	2	0.0007	3.1420	0.0094	0.0024	0.0895
Arginine biosynthesis	1	0.2234	0.6509	1.0000	0.2702	0.0761
Sphingolipid metabolism	3	0.0001	3.8313	0.0024	0.0012	0.0690
Arginine and proline metabolism	1	0.2234	0.6509	1.0000	0.2702	0.0579
N-Glycan biosynthesis	1	0.0063	2.1988	0.0633	0.0134	0.0573
Tryptophan metabolism	1	2.65E-07	6.5762	4.51E-06	4.51E-06	0.0390
Glycosylphosphatidylinositol (GPI)-anchor biosynthesis	1	0.3222	0.4919	1.000	0.3222	0.0040
Biosynthesis of unsaturated fatty acids	1	0.0002	3.6728	0.0032	0.0012	0.0000
Ether lipid metabolism	1	0.0009	3.0653	0.01039	0.0024	0.0000
Arachidonic acid metabolism	1	0.0011	2.9531	0.0123	0.0027	0.0000
Aminoacyl-tRNA biosynthesis	2	0.0129	1.8886	0.1163	0.0244	0.0000
Phenylalanine metabolism	1	0.1328	0.8767	0.9298	0.2053	0.0000
Steroid hormone biosynthesis	1	0.1893	0.7229	1.0000	0.2682	0.0000
Phenylalanine, tyrosine and tryptophan biosynthesis	1	0.2543	0.5947	1.0000	0.2702	0.0000

Table S5. Results of pathway analysis (Placebo pre- vs post-intervention) with MetPA system (MetaboAnalyst 5.0).

Pathway name	Hits	Raw p	-log(p)	Holm adjust	FDR	Impact
Sphingolipid metabolism	4	3.89E-05	4.4098	0.0006	0.0003	0.2475
Glycerolipid metabolism	1	0.0111	1.9552	0.0776	0.0161	0.2368
Arginine and proline metabolism	2	0.0057	2.2465	0.0567	0.0110	0.0919
Arginine biosynthesis	1	0.0062	2.2097	0.0567	0.0110	0.0761
Glycerophospholipid metabolism	1	0.0192	1.7177	0.0835	0.0219	0.0470
Tryptophan metabolism	2	4.87E-05	4.3121	0.0007	0.0003	0.0390
Amino sugar and nucleotide sugar metabolism	1	0.0757	1.1207	0.0835	0.0757	0.0219
Purine metabolism	2	0.0015	2.8383	0.0174	0.0046	0.0167
One carbon pool by folate	1	0.0168	1.7771	0.0835	0.0206	0.0159
Glutathione metabolism	2	0.0006	3.2390	0.0075	0.0023	0.0143
Tyrosine metabolism	1	0.0021	2.6770	0.0231	0.0056	0.0067
Folate biosynthesis	1	0.0167	1.7771	0.0835	0.0206	0.0043
Arachidonic acid metabolism	1	2.93E-05	4.5330	0.0005	0.0003	0.0000
Aminoacyl-tRNA biosynthesis	1	0.0062	2.2097	0.0567	0.0110	0.0000
Galactose metabolism	1	0.0111	1.9552	0.0776	0.0161	0.0000
beta-Alanine metabolism	1	0.0339	1.4703	0.08359	0.0361	0.0000

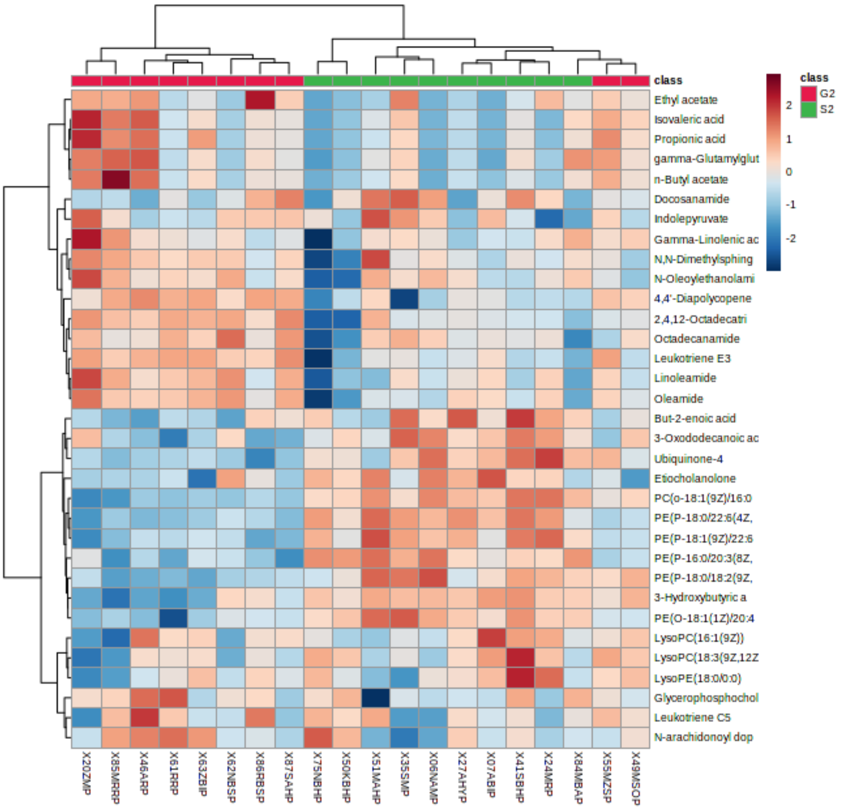

Figure S1. Heat map of the identified plasma metabolites of individuals in IgCo supplemented group and placebo group after 12 weeks of intervention.
